# Innovative problem solving in macaws

**DOI:** 10.3758/s13420-020-00449-y

**Published:** 2020-12-07

**Authors:** Laurie O’Neill, Rahman Rasyidi, Ronan Hastings, Auguste M. P. von Bayern

**Affiliations:** 1 Max Planck Institute for Ornithology, 82319 Seewiesen, Germany; 2 Max Planck Comparative Cognition Research Station, Loro Parque Fundacion, 38400 Puerto de la Cruz, Tenerife Spain; 3 Behavioural Ecology Group, Department of Animal Sciences, Wageningen University and Research, Wageningen, the Netherlands; 4 Department Biology II, Ludwig-Maximilians-University of Munich, Martinsried, Germany

**Keywords:** Comparative cognition, Tool use, Parrot cognition, Causal understanding, Innovation, Physical cognition

## Abstract

Behavioural innovations with tool-like objects in non-habitually tool-using species are thought to require complex physical understanding, but the underlying cognitive processes remain poorly understood. A few parrot species are capable of innovating tool-use and borderline tool-use behaviours. We tested this capacity in two species of macaw (*Ara ambiguus*, n = 9; *Ara glaucogularis*, n = 8) to investigate if they could solve a problem-solving task through manufacture of a multi-stone construction. Specifically, after having functional experience with a pre-inserted stick tool to push a reward out of a horizontal tube, the subjects were required to insert five stones consecutively from one side to perform the same function as the stick tool with the resulting multi-component construction. One *Ara glaucogularis* solved the task and innovated the stone construction after the experience with the stick tool. Two more subjects (one of each species) did so after having further functional experience of a single stone pushing a reward out of a shortened tube. These subjects were able to consistently solve the task, but often made errors, for example counter-productive stone insertions from the opposing end, even in some of the successful trials. Conversely, multiple trials without errors also suggested a strong goal direction. Their performance in the follow-up tasks was inconclusive since they sometimes inserted stones into un-baited or blocked ‘dummy tubes’, but this could have been an attention-deficit behaviour as subjects had not encountered these ‘dummy tubes’ before. Overall, the successful subjects’ performance was so erratic that it proved difficult to conclude whether they had functional understanding of their multi-stone constructions.

**Supplementary Information:**

The online version contains supplementary material available at 10.3758/s13420-020-00449-y.

## Introduction

In the field of animal cognition, problem-solving tasks often involve tool use (Auersperg, Huber, & Gajdon, 2011; Auersperg, von Bayern, Gajdon, Huber, & Kacelnik, 2011; Bird & Emery, 2009; Girndt et al., 2008; Köhler, 1917; Martin-Ordas Call, & Colmenares, 2008, 2012; Mulcahy & Call, 2006; Taylor, Hunt, Holzhaider, & Gray, 2007; Visalberghi & Limongelli, 1994; Visalberghi & Trinca, 1989; von Bayern, Danel, Auersperg, Mioduszewska, & Kacelnik, 2018; Weir & Kacelnik, 2006). Typically, these tool-use tasks present subjects with a novel apparatus that requires some sort of behavioural innovation to access a reward. They are often used as a measure for an individual’s behavioural flexibility and examine what a species understands about the functional properties of those tools, and physical interactions in their environment in general. Of particular interest is comparing the performance of species that are habitual tool users in the wild and those that are not, investigating both closely and distantly related species (Auersperg et al., 2011; Auersperg, von Bayern, Gajdon et al., 2011; Lambert et al., 2017; Teschke et al., 2013). In this manner it is possible to examine if the capacity for flexible tool use is a result of a more general cognitive capacity evolved in response to selection pressures not restricted to the physical domain (e.g., general intelligence; Burkart et al., 2017), or whether more flexible tool-using skills have evolved from a more specialised physical-cognition domain (Kacelnik, 2009; McCormack et al., 2011; Taylor & Gray, 2014).

Tool use, however, is not the only type of physical environmental interaction that might require complex physical cognition. Many species of birds face similar complex object manipulations when they are also required to probe, weave and manipulate many different objects, including sticks, in order to build their nests (Breen et al., 2016). Furthermore, there are many types of extractive foraging without tool use that can require complex manipulations, for example when Gorillas prepare thistles in order to eat them they must manipulate them in such a way so as to avoid being hurt by spines (Byrne et al., 2001). In sum, tool use is not the only measure of an individual’s physical cognition skill.

In this experiment we studied two species of parrots, specifically the macaws *Ara ambiguus* (great green macaws) and *Ara glaucogularis* (blue-throated macaws). Some species of parrots have been recorded as having complex and flexible physical problem-solving skills (Lambert et al., 2018) and they share a number of other life-history and social factors with other taxonomic groups, such as corvids and primates, that suggest they might be required to use complex cognition (Emery & Clayton, 2004; Osvath et al., 2014; Van Horik et al., 2012). These factors include having relatively large, neuronally dense brains that mean they might have a higher upper limit in cognitive capacity (Gutiérrez-Ibáñez et al., 2018; Herculano-Houzel, 2017; Iwaniuk et al., 2005; Olkowicz et al., 2016), although this does not determine or imply high cognitive ability. Many parrots are capable of a diverse range of motor actions, which is likely to be a key factor for behavioural innovations (Griffin et al., 2014), and they have particularly impressive manipulative skills with both their beaks and tongues as well as zygodactyl feet that allow them to hold and manipulate objects in them (Toft & Wright, 2016). Parrots also live in stochastic environments that might require behavioural flexibility in order to thrive (Toft & Wright, 2016), and further to this, many different species of parrots have been shown to be successful invaders to their non-native range (e.g., Menchetti et al., 2016), something that has also been suggested to require elements of behavioural flexibility (Sol et al., 2002, 2005). In total, all the factors of big brains, capable physical manipulation skills and a capacity to respond to changing environments, suggest that parrots might be candidates for using cognitively complex problem-solving skills, such as causal understanding, in order to be adapted to their environment (Van Horik et al., 2012).

A few species of parrots have shown capacities to handle objects in a variety of interesting ways, some of which are borderline cases of tool-use. Vasa parrots (*Coracopsis vasa)* are able to use stones to scrape shells in order to obtain calcium powder for consumption (Lambert et al., 2015). Both hyacinth macaws (*Anodorhynchus hyacinthinus*) and great green macaws (*Ara ambiguus*) have been observed wrapping certain nuts with leaves, which was interpreted as a method to grip the nuts better (Borsari & Ottoni, 2005) or perhaps to avoid the bitter taste of certain piths in their outer layers (Villegas-Retana & Araya-H., 2017). Palm cockatoos’ (*Probosciger aterrimus*) use sticks as part of their mating display, drumming them against hollow trees (Heinsohn et al., 2017; Wood, 1984). Many parrots can face other types extractive foraging difficulties such as trying to manipulate and wield very large and tough-shelled nuts, which require complex manipulations in order to be able to open them (Tella et al., 2015).

Two parrot species in particular have been the focus of physical cognition abilities in parrots and have been shown to be able to use sticks as extractive foraging tools in a number of problem-solving tasks. Kea are able to use stick tools by inserting them into holes in puzzle boxes to knock food items off a pedestal (Auersperg et al. 2011; Auersperg, von Bayern, Gajdon et al. 2011), push food items out of a tube (Lambert et al., 2017), and even to spring traps meant for other animals in the wild in order to retrieve the bait (Goodman et al., 2018). Goffin’s cockatoos have been established as probable non-tool users in the wild (O’Hara et al., 2019; but see Osuna-Mascaró & Auersperg, 2018), but remarkably some individuals began to spontaneously innovate the manufacture and use of stick tools in captivity (Auersperg et al., 2012). Furthermore, these cockatoos appeared to show understanding of some of the complex properties of these tools that made them functional, such as making tools of the appropriate length (Auersperg et al., 2018) or adding hooks to their tools when necessary (Laumer et al., 2017). Because of these examples, it is interesting to expand tool-use tasks to other species across the parrot order. The Kea and Goffin’s cockatoos are members of the more basal parrot superfamilies *Strigopoidea* and *Cacatuoidea*, respectively, whereas the macaws in this study are parrots of the family *Psittacoidea,* so the current experiment provides an opportunity to expand our understanding of parrots’ physical cognition capacities across the parrot order. We can therefore explore what kind of understanding these various species have for tools and instrumental problem solving, and whether there are certain evolutionary factors that might drive more complex physical cognition abilities.

The specific individuals we tested have faced tool-use tasks before. In a single trial-test of a ‘food-out-of-reach’ situation with a stick tool available, they did not interact with a stick tool (Krasheninnikova et al., 2019). In the stone-dropping task, a problem-solving task that requires individuals to innovate the use of a stone tool to get a reward (von Bayern et al., 2009), the majority were capable of using stone tools to drop onto a collapsible platform to retrieve a reward, but most individuals were unable to manipulate a stick tool to perform a very similar action (O’Neill et al., 2020). As some species of macaws have shown borderline tool use abilities in the wild before (Borsari & Ottoni, 2005; Villegas-Retana & Araya-H., 2017), we believed them to be suitable candidates to test the functional understanding of instrumental problem solving in the lab.

The inspiration for the experimental task we used comes from Visalberghi and Trinca (1989). In that experiment, a group of tufted capuchins (*Sapajus apella)* were given a transparent, long, horizontal tube, with a reward in the middle. To solve the task they had to insert a stick tool into one side of the tube and push the reward out of the other side. After successfully doing this, they were given the tube apparatus again but only with access to three smaller sticks, none of which could reach the reward on its own. The subjects had to insert two of these smaller sticks, one behind the other, in order to push the reward far enough to the other end of the tube so that it was accessible. The aim of the task was to see if the subjects recognised the functional property of the stick tool that they initially inserted, or whether they were just ‘performing’ a learned action of ‘insert sticks’ as this would lead to rewards. The modification we made on this task was to simply replace these small sticks with even smaller stones. Thus, more of these stone tools would have to be inserted into the tube before the reward was available (a minimum of four). This behaviour could possibly be classed as an ‘additive tool’, as the subjects would need to “*join or connect two or more objects to make one tool that is held or directly manipulated in its entirety during its eventual use”* (Shumaker et al., 2011). However, it is not certain whether this multi-component structure would fit all definitions of tool use (e.g., Fragaszy & Mangalam, 2018). Nevertheless, the focus of the experiment was not ‘whether the subjects could use tools’, but instead it was to explore subjects’ causal understanding of the underlying functional properties of the initial stick tool they interacted with to see if they could recreate the functional properties in another way. For this reason, we do not refer to the multi-component construction subjects were manufacturing as an additive tool, as we recognize this is not a name that everyone will accept. Instead, we refer to it as a ‘multi-stone construction’ or just a ‘stone construction’.

One issue with this task is that the capuchin subjects appeared to be able to find a solution through exploratory, trial-and-error behaviour (Visalberghi & Trinca, 1989). These kinds of persistent behaviours have been noted to predict many animal subjects’ ability to solve problem-solving tasks in other experiments (Chow et al., 2016; Thornton & Lukas, 2012; Thornton & Samson, 2012; van Horik & Madden, 2016). Specifically, the three capuchin subjects that were able to solve this additive tool task repeatedly made an error of inserting a stick from both sides of the reward before inserting a stick behind one that had been previously inserted (and thus solving the task). This suggested that the subjects did not have an end-state mental representation of the multi-tool construction they were making with the shorter sticks (e.g., Jelbert et al., 2018), and instead were just randomly inserting sticks until they could reach the reward. Nevertheless, the subjects were still able to solve the task after making this error, as the apparatus only needed two stick insertions from one side to reach the reward. This may have meant there was little motivation for the example capuchin subjects to optimise their multi-tool construction, as there was little to no negative outcome from the errors they made. To solve this issue in our replication of the experiment, we required that there were at least four insertions of a small stone from one side before the reward was available from the other. Additionally, they also typically required three consecutive actions before they had visual feedback that the reward was being moved.

**Fig. 1:**
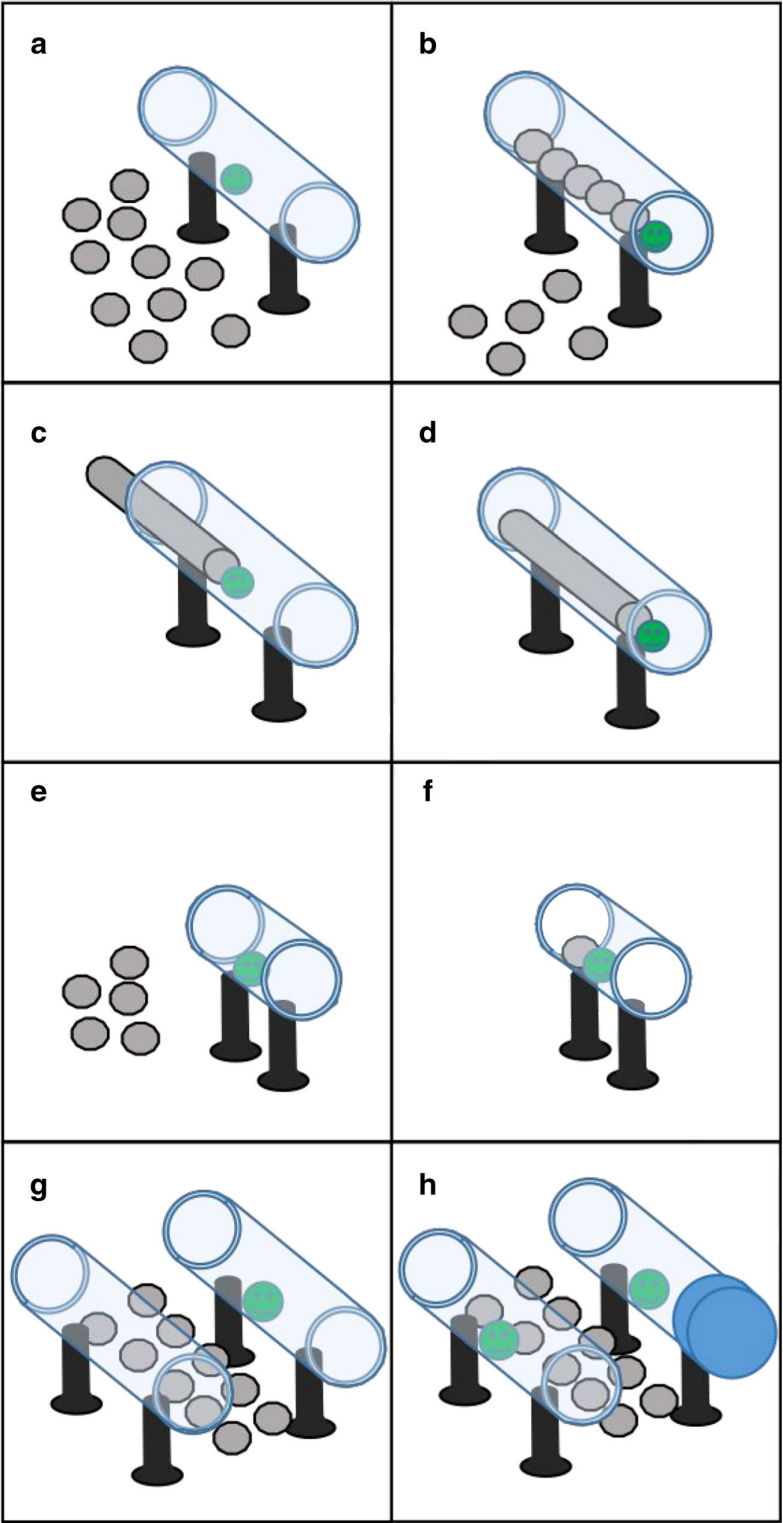
In the pre-test and critical tests a reward (a walnut represented by a green circle in the figures) was placed in the middle of a transparent acrylic tube with ten stones placed around it (**a**). In the **Pre-test** and **Critical tests,** subjects had to insert five of these into one side of the tube to push out rewards on the opposite end (**b**). In the **stick experience** phase, a stick was inserted into the side of the tube up until the reward (**c**). Subjects had to push the stick to push the reward out (**d**). In the **short-tube experience** a smaller tube was given to the subjects in which only one stone needed to be inserted into the tube and pushed to make the reward come out (**e**). In the **pre-inserted stone** experience, a stone was inserted to be touching the reward in the short tube, so that the stone only needed to be pushed to make the reward come out (**f**). In the **parallel tubes** phase, there were two tubes, but only one of them was baited (**g**), subjects had to target the stones only at the baited tube. In the **blocked tubes** phase, both tubes were baited, but a bung prevented the reward from being removed from one tube (**h**). For clarity in the schematics, a vertical tube in the middle of the apparatuses is not shown. This was featured in all apparatuses except the short tubes (**e** and **f**). This vertical tube can be seen in the supplementary videos showing the subjects interacting with the horizontal tube

The rationale of the experiment was as follows. The first stage of the experiment, the *pre-test*, gave the subjects an opportunity to spontaneously solve the task and see if they were able to insert multiple stones, one behind the other, to push a reward out of a horizontal tube (Fig. 1a and b). It was expected that they would not be able to do so, thus after this phase the subjects were given an example of the end-state function of the multi-stone construction they were required to make with the stones, ergo a stick was pre-inserted into the tube and the subjects simply had to push it to remove the food (Fig. 1c and d). After this, the subjects were once again given the opportunity to solve the task with the stones – *critical test 1* (a repeat of Fig. 1a and b). The underlying principle was that if subjects were able to reason the underlying functional mechanism that made the stick effective, then they would be able to recreate the same effect of this stick out of multiple stones. If the subjects were still unable to solve the task at this stage, then they were given a more direct experience of the efficacy of the stones, thus cueing them to use stones. They were given a similar apparatus, but the horizontal tube was shortened and only a single stone needed to be inserted and pushed to make the reward available on the other side (Fig. 1e and f). If they succeeded in doing that then subjects were given another critical test (*critical test 2*) to see if they could chain this single stone insertion behaviour repeatedly to make the required multi-stone construction. In essence, in each of the three test-phases (the *pre-test* and *critical tests 1 and 2*), subjects had to make a smaller innovative ‘leap’, and use an assumed ‘less complex’ form of cognition, to solve the task. After the subjects were able to consistently solve the task, we also gave them up to two additional follow-up tasks in order to triangulate (Heyes, 1993) how much the subjects understood about the function of their multi-stone construction (Fig. 1g and h).

Although the subjects we tested were naïve to the specific apparatus that we gave them in this experiment, they were not naïve to the experimental protocols in general. For this reason we decided to exploit their testing history to also elucidate what kind of heuristics (Hutchinson & Gigerenzer, 2005) and behaviours the subjects may extend from successful behavioural interactions in previous experiments in order to find rapid innovations to the novel apparatus in this experiment, a mechanism thought to be vital in animal innovations (Tebbich et al. 2016). We therefore added a ‘red-herring’ to the apparatus. Specifically, this was in the form of a vertical tube, attached to the horizontal tube, that was placed directly over the reward, but was blocked and so had no connection to the reward (see Supplementary Fig. 7 (Online Supplementary Material, OSM) for a photo of the apparatus). Placing a rock into this vertical tube would therefore achieve nothing. Nevertheless, in the previous experiment they completed, the stone-dropping task (O’Neill et al., 2020), the solution to the experiment was to place a stone tool into a vertical tube. Thus, we could see if they would apply this same rule in their exploration of a new apparatus that was drawn from success in a previous experiment.

We predicted that at least some of the parrots would be likely to solve the task, as we have seen previously that the subjects were highly explorative and capable in this similar stone-dropping problem-solving task (O’Neill et al., 2020). However, we were not certain whether the subjects would be able to solve the task consistently nor succeed in the transfer tasks as this experiment required more self-control, something we knew the subjects might lack (Kabadayi et al., 2017).

## Methods

### Subjects and housing

Nine great green macaws (*Ara ambiguus*) and eight blue-throated macaws (*Ara glaucogularis*) were tested. Their age and sex are shown in Table 1. All the birds were hand-raised and group-reared by the Loro Parque Foundation in Tenerife, Spain, and all were housed in the Comparative Cognition Research Station, within the Loro Parque zoo in Puerto de la Cruz, Tenerife. The birds were housed in groups of two to six individuals, according to species and age, in seven aviaries. Six of these aviaries were 1.8 × 6 × 3 m (width × length x height) and one was 2 × 6 × 3 m. Windows of 1 × 1 m could be opened between the aviaries to connect them together. One half of each aviary was outside, so the birds followed the natural heat and light schedule of Tenerife. The half inside the research station was lit with Arcadia Zoo Bars (Arcadia 54W freshwater Pro and Arcadia 54W D3 Reptile Lamp) that followed the natural light schedule.

**Table 1:**
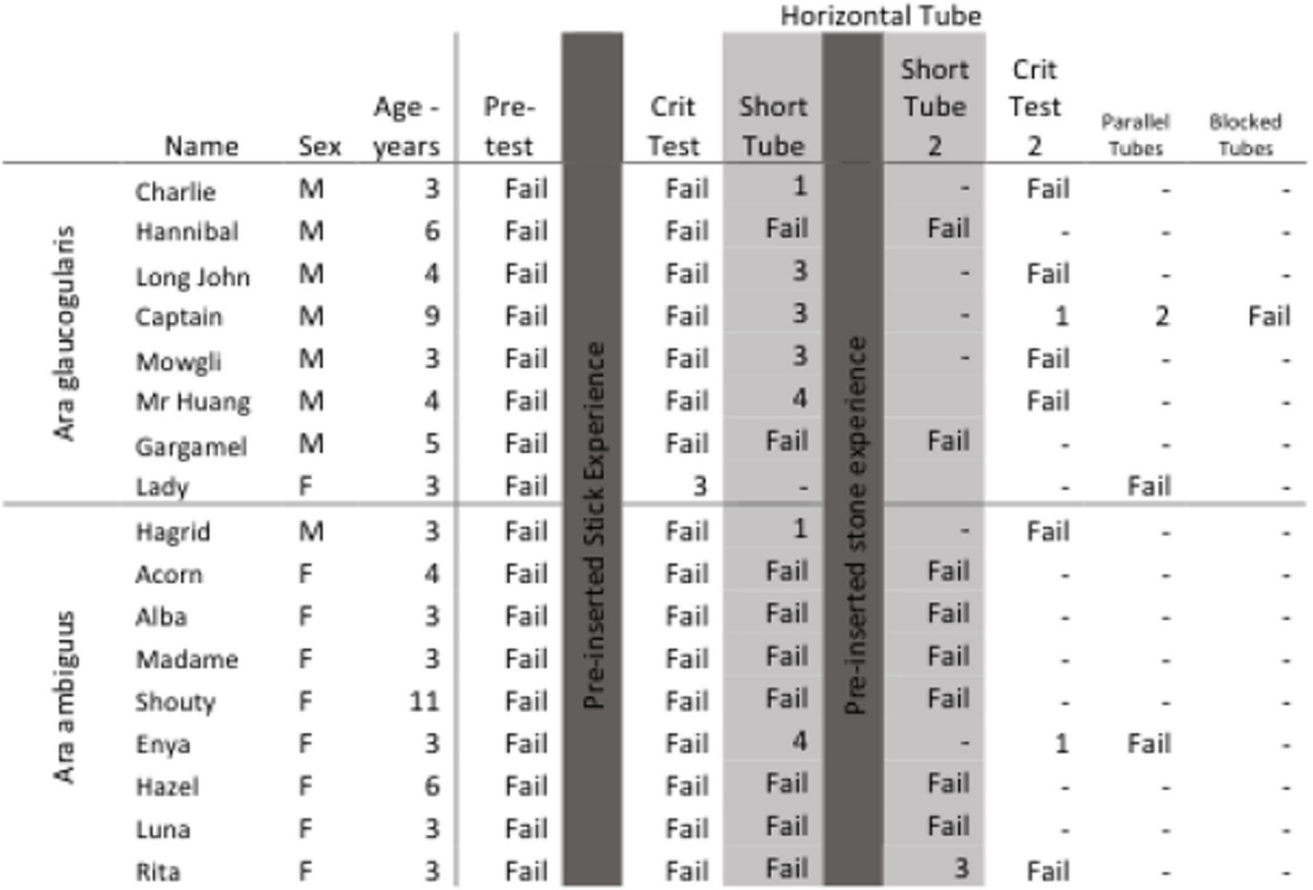
The age, sex and success of each subject taking part in the *horizontal tube task* The table also shows which stage of the experiment each individual completed and at which stage they failed or completed the task. The numbers in the columns show the session in which the subjects first had a successful trial in that particular test phase. Dashes indicate that the subject did not take part in that test phase. Columns in both shades of grey are the *mechanistic experiences*

A large variety of fresh fruit and vegetables were provided first thing in the morning and again in the afternoon. In the afternoon, each individual received a portion of Versele Laga Ara seed-mix that was modified based on their daily weight. The parrots were never starved, and portions of nuts and seeds were controlled to prevent overeating and obesity. Testing took place at least an hour after the parrots had breakfast to ensure there was sufficient motivation to get food rewards. Unless otherwise specified, walnut halves were used as rewards. These were highly prized by all subjects, and they were able to obtain them on a daily basis through voluntary testing.

### Experimental rooms

Experiments took place in separate testing rooms away from the aviaries. All birds had been previously trained to enter them. These rooms were 1.5 × 1.5 × 1.5 m and also lit with Arcadia Zoo Bars to cover the birds’ full visual range. One wall of each one of the experimental rooms had a 50 × 25 cm window through which an experimenter could place apparatuses into the testing room from a neighbouring chamber. This window could be occluded with a white board so that the experimenter could hide anything they were doing from the subjects’ view, such as re-setting apparatuses between trials. The experimenter always wore mirrored sunglasses during experiments to prevent their gaze being a cue for the parrots. A second wall was made of sound-proofed one-way glass so that zoo visitors could observe experiments without disturbing the subjects.

One individual, Hannibal, was tested in a separate cage connected to his home aviary as he was not comfortable going to the experimental rooms. Each aviary had a separate, removable cage attached to it, which was used to transport subjects between their home aviaries and the experimental rooms. However, the cages were also large enough for testing (1 × 1 × 1 m). They had sliding doors that could separate an individual from the rest of their aviary, and were also on wheels, which meant they could be moved to an area occluded from the other individuals in their aviaries. Finally, they also had a second door through which testing apparatuses could be placed into the cage with a test subject. In this way, Hannibal was able to complete the test in a similar manner to the other subjects.

### Apparatus

This task required subjects to push a reward out of a horizontal transparent tube, which was 30 cm long with a diameter of 6 cm (Fig. 1a and b). The tube was fixed in an elevated horizontal position at a height of 10 cm by a metal stand at either end (see Supplementary Fig. 7 (OSM) for picture). The stands were attached to a solid wooden board (45 × 30 × 2.5 cm) and the central tube was wrapped in wire mesh (1 × 2 cm) to show the solidity of the transparent acrylic. The reward was placed in the middle of the horizontal tube, thus the only way to obtain the reward was by pushing it to one of the two edges with the aid of a tool. There was an additional 5-cm tube (vertically positioned) of the same diameter attached to the centre of the top-side of the main tube (see Supplementary Fig. 7 (OSM)). This additional tube was non-functional as no tool could interact with the reward from here. Its purpose was to provide a place for the subjects to put the stone tools onto the top of the apparatus. This was so that the subjects’ tendency and persistence for placing tools in an opening on top of the apparatus could be assessed, even when it had no effect on the outcome of the task.

For each test, the subjects were provided with a selection of ten stones. All were natural volcanic rocks taken from the beach in Puerto de la Cruz, Tenerife (they were returned to the beach after testing was finished). They all fit into the tube, had a diameter of 4.5–5.5 cm and weighed between 60 and 100 g. Three stones had to be placed into the tube, one behind the other, before the reward moved. At least four stones had to be inserted to place the reward within reach. This meant that the subject received no perceptual feedback of the reward moving until at least three specifically directed actions were made.

### Experimental procedures

**Fig. 2:**
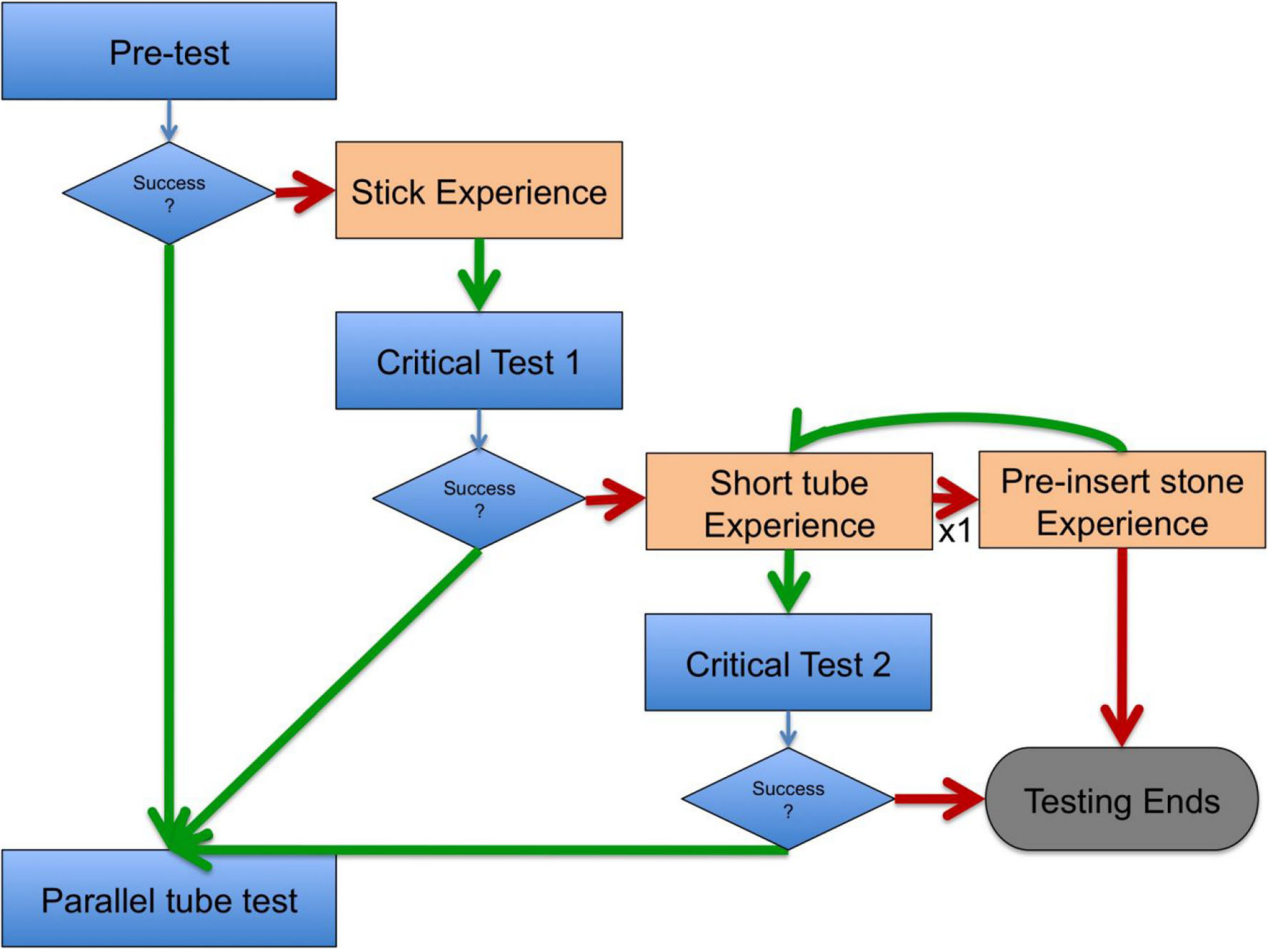
The subjects were given progressively more functional experience if they failed to solve the task. In blue are the ‘test’ phases, in which subjects were exposed to the task that required them to use a multi-stone construction to push the reward out of the tube. In orange are the ‘experience’ phases, which provided the subjects with mechanistic experience of how the task could be solved. If subjects succeeded in a phase they followed the green arrow, if they failed then they followed the red arrow. Subjects that failed the short-tube experience moved onto the pre-inserted stone experience, if they succeeded in the preinserted stone experience they were given the short-tube experience again. If they failed the short-tube experience a second time then testing ended, i.e. they only had the pre-inserted stone experience once

The experiment consisted of three test phases (*pre-test, critical test 1* and *critical test 2*), three different experience phases (*pre-inserted stick, short tube 1, pre-inserted stone* and *short tube 2*) and two transfer tasks (*parallel tubes* and *blocked tubes*). An overview of the order in which these phases took place is shown in Fig. 2. Not every subject took part in every phase as they either skipped phases due to early success in an experimental phase or did not take part due to failure in an experience phase.

In essence, in the pre-test the subjects were given an opportunity to spontaneously solve the task in order to examine if the experiment could be solved without any mechanistic experience, just by exploration behaviours characteristic of the parrot subjects. The experience phases presented the subjects with progressively more specific direct mechanistic experience of how the experiment could be solved: the first mechanistic experience (*pre-inserted stick)* provided them with knowledge of the basic mechanism that the food could be pushed from contact with a stick, but without cueing them towards stones in particular. The second mechanistic experience (*short tube*) allowed the subjects to move the reward by just inserting a single stone, thus reinforcing subjects that showed exploratory stone-inserting behaviour by letting them experience that their behaviour can move the reward and lead to success. The third mechanistic experience further helped subjects that would not insert stones (*pre-inserted stone*) as it showed that just pushing stones already pre-inserted in the tube would move the reward, hence cueing them both towards stones and the opening of the tube. The critical test phases, after each of these mechanistic experience phases, were implemented to test if the subjects could generalise and extrapolate from these progressively more specific mechanistic experiences to produce the necessary actions to solve the task. Finally, the subjects were offered the parallel and blocked tubes tasks as follow-up tasks to investigate if they could flexibly solve slightly modified tasks, verifying whether they indeed attended to the functional properties of the task or not.

Within the protocol descriptions, the term ‘session’ describes a single period of testing in the experimental chamber. Typically subjects took part in a single session per day. Within each testing session (pre-test, both critical test phases, and the parallel and blocked tubes phases), subjects could be given a maximum of three trials (i.e. chances to solve the task and obtain a reward). Within each session in the mechanistic experience phases (pre-inserted stick, short tube and pre-inserted stone), subjects could be given a maximum of six trials. This was because the successful method in the test sessions took slightly longer and we did not want subjects to lose motivation. If subjects failed a trial, the whole session was stopped and subjects were brought back to their group aviary. A trial was considered as failed if subjects did not solve the task within 10 min of the apparatus being placed into the experimental room with them. If subjects succeeded in a trial, then the apparatus was removed, re-baited and replaced into the subject’s experimental room for another trial (described below), unless it was the last trial of a session, in which case the session ended. All subjects finished testing, from habituation to final test trial, within 2 months. All testing took place between November 2017 and May 2018.

#### Habituation

Every subject was first habituated to the apparatus without the presence of the stones. These sessions took place in the experimental rooms, with an experimenter sitting in the neighbouring chamber. This was necessary for re-baiting if further trials were needed in the session and it also served to create a more relaxing environment for the subjects to work in. Once the subject was in the experimental room, the experimenter introduced the apparatus through the window in between them. Subjects had 5 min to approach the apparatus and take a piece of walnut placed at the base of the apparatus. If they took it, the experimenter waited for 30 s then removed the apparatus. Next, the experimenter, out of sight of the subject, re-baited the apparatus and reintroduced it 1 min after removing it. The subjects had up to six of these trials per session and if they took six walnut pieces in a row within a single session, they moved onto testing.

The subjects had already been habituated with the stone tools in a previous experiment and all were eager to interact with them, so they did not need habituating to them again. We state that the parrots were ‘eager’ to interact with the stones because they would frequently pick up the stones and just manipulate them between their feet and beaks with no apparent purpose. This was typical for these macaws for any object that was a similar size and shape to these stones.

#### Pre-test and critical test procedures

The pre-test and both critical test procedures were identical. The difference was that subjects had been given different mechanistic experience phases after the *pre-test* and between *critical tests 1 and 2*, which provided functional information about how the task could be solved. The order of these tests and mechanistic experience phases can be seen in Fig. 2.

Subjects were given a minimum of six 10-min sessions for both the pre-test and the two critical tests. Each session had between one to three trials, i.e. they were given more trials in a session if they succeeded until the maximum trial number of three was reached. The apparatus was first prepared out of sight of the subjects. A half walnut reward was placed inside the middle of the tube and ten of the stones were placed on the board beneath it. The whole apparatus was pushed into the subject’s chamber, in the centre of the testing area; thus the subjects were able to access it from all sides. The subjects were given a maximum of 10 min to interact with the apparatus. If they failed to retrieve the reward in this time, then the trial and the session ended, the apparatus was removed, and the subject was returned to its social group. If, on the other hand, the subject managed to successfully retrieve the reward by pushing multiple stones into the side of the apparatus, then the subject was given further trials within that testing session. To do this, the experimenter waited for 30 s after the subject had retrieved the reward and then removed the apparatus. It was re-baited with another half piece of walnut out of sight of the subject, ensuring that there were ten stones available underneath the apparatus. The apparatus was then placed back into the room with the subject for another trial exactly 1 min after it was removed from the subject’s chamber. Subjects received a maximum of three trials in a session. They needed to complete 12 successful trials to be considered consistently successful within this experimental phase (although Captain and Enya, see Table 1, progressed from the second critical test phase with 11 successful trials due to an experimenter’s error). This repetition was to validate that subjects had not accidentally succeeded in a task but could replicate the method they used to get the reward. Additionally, if subjects had six failed trials in a condition, they moved onto the next mechanistic experience phase (or stopped testing, depending on which condition they had ‘failed’; see Fig. 2).

Sessions were only counted as valid if the subjects approached and touched the apparatus. Thus unsuccessful trials were ones in which subjects had touched the stone tools or the apparatus but had not been able to retrieve the reward. Subjects that were able to succeed in the pre-test or either of the critical test phases moved onto the follow-up tasks (*parallel tubes* and *blocked tube*).

#### Pre-inserted stick experience

In the first mechanistic experience phase, called *pre-inserted stick experience*, a stick tool was used to show how the reward could be pushed out of the tube (Fig. 1c and d). In the first stage of this phase, the stick tool was attached to the tube so that it could only be pushed into the tube, i.e. it could not be pulled out. We assumed the parrots would be likely to pull the stick tool in their initial exploratory interactions with it, so attaching it in this way made sure they could only experience the ‘rewarding’ outcome of pushing the stick first. The experimenter placed a half walnut reward in the centre of the apparatus with the stick tool inserted flush against it. The subjects were given multiple 10-min sessions to push the stick through explorative behaviour alone. If they succeeded within a session, the experimenter gave the subjects more trials. They removed the apparatus, re-baited it out of sight, ensured the stick tool was correctly positioned and gave it back to the subject. If subjects successfully obtained the reward six times within a session, they moved onto the next stage of this mechanistic experience phase.

In this following stage, the procedure and setup were identical, except the stick was no longer attached to the apparatus, so it was possible for the birds to pull the stick out of the apparatus. We did this to ensure that the subjects recognised that the stick specifically needed to be pushed to get the reward, and it wasn’t just exploratory interaction with the stick that led them to obtaining the reward. If subjects pulled and thus removed the stick at this stage, the experimenter would remove the apparatus and the stick, reposition the stick inside the apparatus out of sight of the subjects, and then give it back. There was no limit to the number of times the stick could be re-positioned like this (but there was still a 10-min time limit per trial). To pass this final stage of the experience phase, the subjects had to push out the reward 12 times in two consecutive sessions (six trials per session). All subjects completed the first stage of the *pre-inserted stick experience* in a maximum of six sessions and the second stage in a maximum of three sessions. They then moved onto the first *critical test*.

#### Short tube experience

If the subjects failed the first *critical test*, they were given a second mechanistic experience phase. In this mechanistic experience phase, called the *short-tube experience*, a modified apparatus was used (Fig. 1e). A 15-cm long tube was mounted on a separate wooden board at the same height as the original apparatus. It did not have the additional vertical tube in the centre. With this shorter tube, the subjects were still unable to reach a reward placed in the middle, but now they only needed to insert one stone to push the reward out of the tube. Some subjects had inserted one or more stones into the apparatus in the initial *critical test*, but not a sufficient number to move the reward (see *Results* section). If they repeated this behaviour with a shorter tube it would be enough to move the reward.

The short tube was given with the same procedure as the *critical test,* i.e. the subjects were given the short-tube apparatus with ten stones and they then had 10 min to solve the task by placing one stone into the tube and pushing the reward out. After successful trials, the apparatus was removed, re-baited, and placed back into the subject’s room. However, as this solution to the short-tube experience phase was potentially very rapid, as the subjects only needed to insert a single stone, subjects were given up to six trials per session to speed up the testing process. To reach the criterion in the *short-tube experience*, and in turn proceed to *critical test 2*, they had to succeed 12 times consecutively over two sessions. If subjects failed a trial in a session, i.e. they did not obtain the reward within 10 min, then the trial and session ended, apparatus was removed, and the subjects were taken back to their social group. If subjects failed six trials in the *short-tube experience*, then they were given another mechanistic experience phase, the *pre-inserted stone* phase.

If the subjects succeeded in the *pre-inserted stone experience*, then they still had to succeed in the *short-tube experience* before they could proceed to *critical test 2*. However, the subjects only had one opportunity to succeed in the *pre-inserted stone experience*, i.e. subjects only moved between the *short-tube experience* to the *pre-inserted stone experience* once, not continually until they succeeded.

#### Pre-inserted stone experience

The *pre-inserted stone* mechanistic experience used the same short tube apparatus (Fig. 1f). However, now one of the stone tools was already placed inside the tube next to the reward so the subjects only needed to push the stone to obtain the reward. The purpose of this was to cue the subjects to the correct positioning of the stone and that interaction with the stone when it was in this position would also make the reward move. If subjects succeeded in this stage, then they were given another chance to succeed in the *short-tube experience,* which they then had to succeed on in order to proceed to *critical test 2*.

In this experience phase the experimenter placed one of the stones in the tube, flush against the reward, before giving it to the subject. This setup was done out of sight of the subjects, so they did not receive a cue from the experimenter to specifically insert the stones into the tube, they only saw the apparatus with a stone already inserted. For each trial, the subjects had up to 10 min to obtain the reward. If the reward was obtained, the apparatus was removed, re-baited, and given back to the subject. If subjects could repeat this behaviour six times in one session, they were given another round in the *short-tube experience* phase, exactly as described above. If they passed criterion in the latter, then they were given *critical test 2*, but if they failed, their participation in the test ended.

All of the subjects that were given the pre-inserted stone phase succeeded (nine subjects), but only one of these subjects (Rita) managed to succeed in the following *short tube 2* experience phase (see Table 1).

#### Parallel tube and blocked tube transfer tasks

Two follow-up tasks were devised for subjects that managed to reliably succeed in one of the critical test phases. The purpose of these was to inform on how the subjects had achieved their successful methods in the critical test phase.

Specifically, the *parallel tubes test* (Fig. 1g) was used to see if subjects were inserting stones into the tubes to move the reward or whether they had just learnt that inserting stones into the tubes was rewarding. In this task, two identical versions of the horizontal tube were attached to a single board, parallel to each other. The key difference was that only one of the two tubes was baited with a walnut reward, the testing protocol was otherwise the same as the *critical test phases*. The subjects were still only provided with ten stones to try and obtain the rewards and they were still given 10 min per trial to try and obtain the reward. If they obtained the reward, the apparatus was removed and re-baited; however, the reward was randomly placed in either of the two different tubes in different trials in a counterbalanced schedule, i.e. rewards were not just swapped between the tubes on different trials but were placed in each of the two tubes an equal number of times over all trials. A more stringent success criterion was used in this phase. Subjects had to obtain the reward 12 times in a row in four sessions (three times per session), i.e. without unsuccessful trials between the successful trials. This was to ensure that random placement of stones would not lead to subjects reaching the success criteria in this phase. If they succeeded, they moved onto the *blocked tube task*, but if they failed, they stopped testing.

The *blocked tube* task used the same apparatus as the parallel tubes, but a ‘bung’ attachment was created to block one end of one of the tubes (Fig. 1h). This transfer task was used to see if the subjects could recognise they needed to push the reward to a location that was then accessible to themselves, not that the rewards just needed to be pushed. In this task, both tubes were now baited, but the bung would prevent the subjects from pushing the reward out of the blocked tube. Between every trial the bung was randomly repositioned in one of the four available positions, again in a counterbalanced fashion. The success criterion would have been the same as the parallel tubes task, but no subject succeeded here. This was the last available task in the experiment.

### Behavioural coding

All experiments were recorded on four static CCTV cameras. These covered all angles of the testing room. Two recordings from separate cameras were saved for each experimental session, but more recordings were saved if it was necessary for specific trials, for example if there was partial occlusion of a camera view from the subject standing in the way. Behaviours were scored from the videos using Solomon coder (András Péter, solomon.andraspeter.com).

Firstly, the location of the placement of the stone tools was recorded. For every trial in the ‘*pre-test’*, ‘*critical test’* and ‘*critical test 2’* the number of stones that subjects placed in each side of the tube was counted. The apparatus was always placed into the testing rooms in exactly the same position and orientation so the locations we recorded of the stones was ‘absolute’, i.e. the left side of the tubes from one specific camera angle was considered ‘side 1’ and the right side was ‘side 2’. We did not call these ‘left and right’ as occasionally a second camera angle was consulted to confirm stone insertions and the other cameras were pointed from different angles, hence left and right may have made this confusing whilst coding. A stone was classified as inserted into the tube if the subjects placed it inside the tube, released it from their beak, and then it stayed inside the tube without falling out. Sometimes, subjects removed stones from the tubes and then placed them back inside the tube, either on the same or on the other side. If the stone was picked up from inside the tube, removed from within the tube entirely, then placed back inside the tube and released, it counted as another insertion. In this manner, it was possible for more than ten stone insertions to be counted per trial even though there were only ten stones available for each trial. In the *parallel tubes* and *blocked tube* transfer tasks, the number of stones inserted on each side of *both* tubes was counted, i.e. there were four possible places where the stones could be inserted. For both of these tasks, one tube was the ‘correct tube’ and the other was the ‘wrong tube’, as rewards could not be obtained from the ‘wrong tube’.

In all of the test trials, from the *pre-test* and *critical tests* to the *parallel tubes* and *blocked tube* transfer trials, we also counted the number of times subjects placed stones into the vertical-tube on top of the horizontal tube. In some trials, subjects again removed and re-inserted stones on multiple occasions into these tubes. The same rule as above was followed: if the stone was picked up, removed entirely from tube, then re-inserted and released, it was counted as another stone insertion. Hence, in some trials many more than two ‘stone-in-top’ insertions were counted even though the vertical tube could only hold two stones at once. In the parallel tube and blocked tube trials, there was no discrimination between subjects placing stones in the top of the ‘correct’ or the ‘wrong’ tube.

Ten percent of the videos (28 sessions, 38 trials; these were randomly selected from all of the pre-test, critical tests and parallel/blocked tubes tests) were then coded by a second observer to check inter-observer reliability on the number of stones inserted into both the top and the sides of the tube. There was ‘excellent’ reliability between the two observers (Intraclass correlation = 93.9% consistency, R-package “irr”).

Finally, we also coded subjects’ exploration of the stones and the apparatus. This included the latency until subjects touched the stones and the apparatus in each trial as well as the amount of time the subjects touched the stones and apparatus in each trial. Finally, the amount of time the subjects spent touching the apparatus *with* the stones (combining the objects together) was also recorded. This data was not used in the final analysis, but a summary of it is provided in Supplementary Table 2 (OSM), and the complete data are with the raw data to be found on figshare (O’Neill & Bayern, 2020).

## Results

**Fig. 3:**
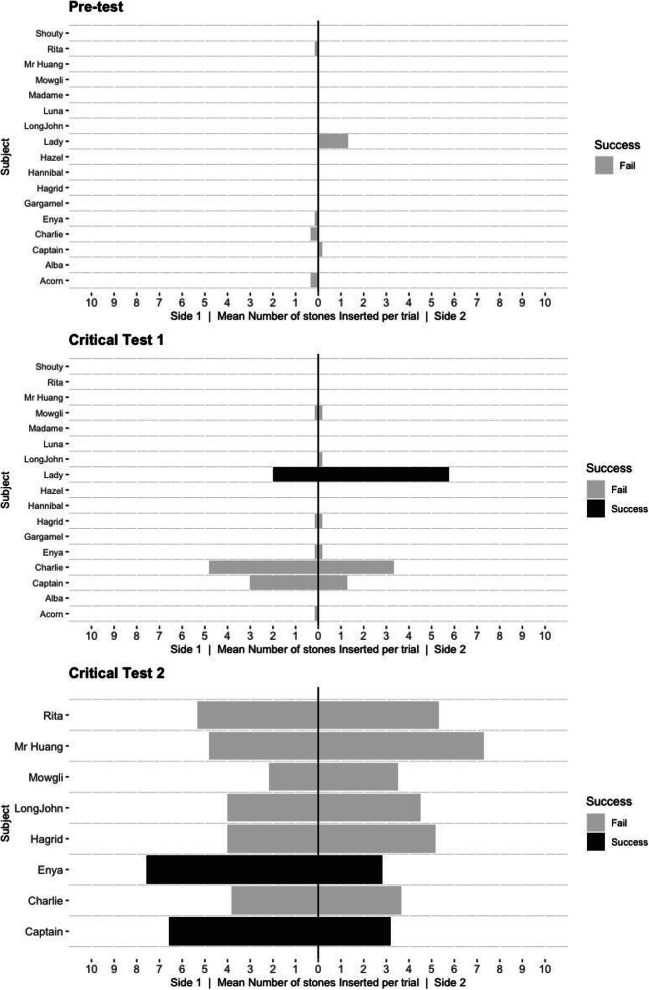
The individual mean number of stone insertions into each side of the tube per trial. Grey bars indicate that individuals failed in that phase of testing, black bars indicate that individuals consistently succeeded in that phase of testing. There are fewer subjects in *critical test 2* as not all individuals passed the *short-tube experience*, which was a requirement to complete *critical test 2*

**Fig. 4:**
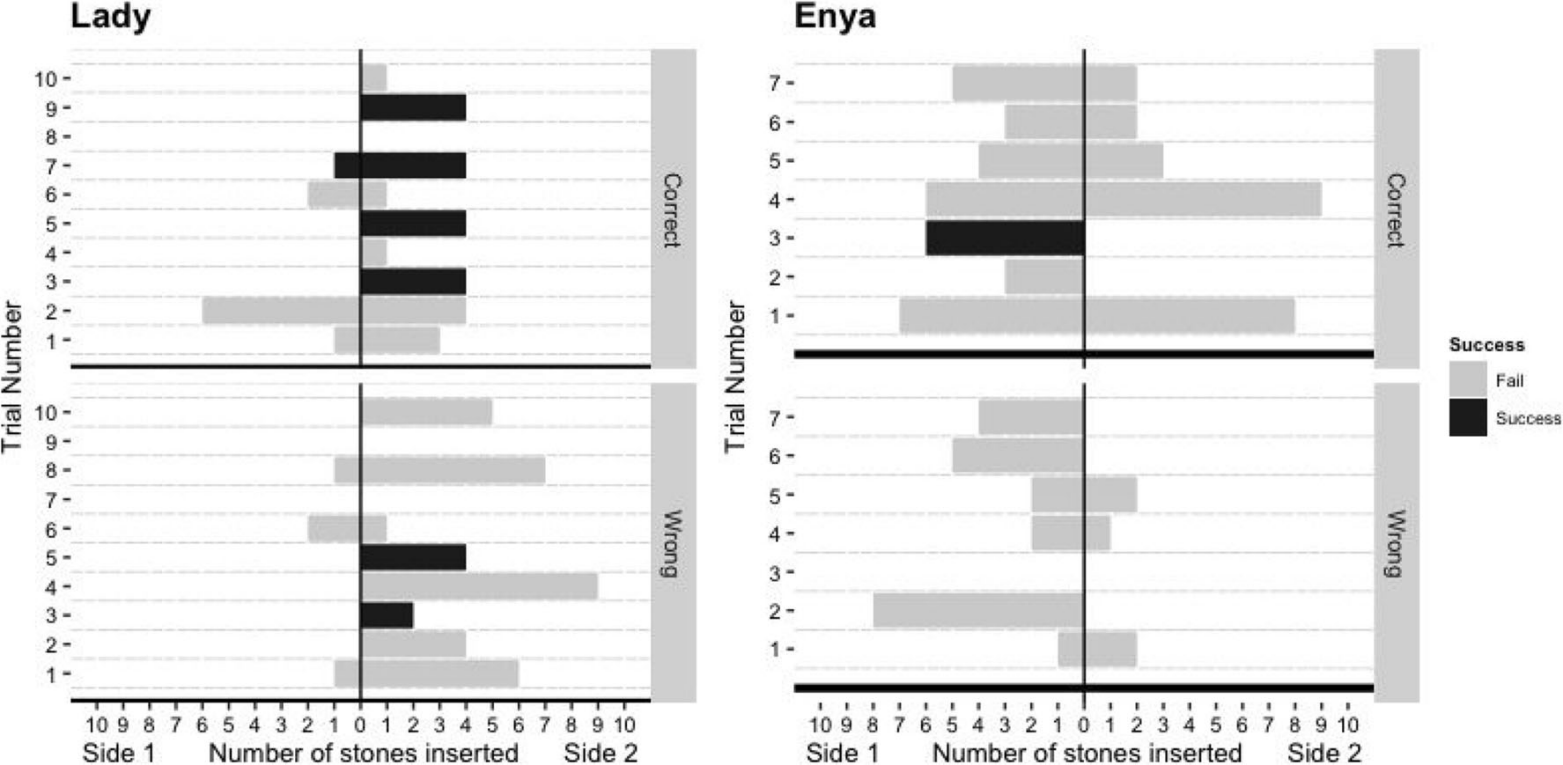
The number of stones inserted on each side of each tube in the *parallel tubes task*. The top chart of each bird shows the stones inserted in the baited tubes, the bottom shows the unbaited tubes. Lady (*Ara glaucogularis*) appeared to insert stones into either of the tubes, but specifically from one direction. Sometimes this meant she inserted stones into the unbaited tube, sometimes it led to success. Enya (*Ara ambiguus*) appeared to start inserting stones more randomly at this stage, so she had very little success

**Fig. 5:**
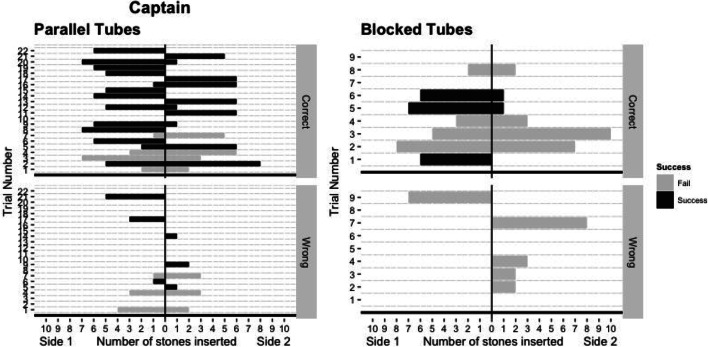
The number of stones inserted by Captain into the ‘correct’ and the ‘wrong’ tube in the *parallel tubes* and the *blocked tube* task. The top chart of each task shows the stones inserted in the baited tubes, the bottom shows the unbaited tubes. Captain mostly avoided the unbaited tube in the *parallel tubes* task, but began to fail trials in the *blocked tube* task by inserting tubes into this baited, but blocked (so the reward was inaccessible), tube. He did not have an absolute side preference in the *parallel tube* task, but had successful trials from inserting stones from either side. At this stage he also had many trials in which he didn’t make the error of inserting rocks from the opposing side which he had started. He did appear to lose motivation in the blocked tube task

In total, three birds (two out of eight *Ara glaucogularis* and one out of nine *Ara ambiguus*) were able to consistently solve the task in the critical tests (Fig. 3). One *Ara glaucogularis* (Lady) managed to do this in *critical test 1* after the *stick experience* phase (Fig. 3; Supplementary Fig. 3 (OSM)); she solved the task 12 times in 16 trials. The other two, Enya (*Ara ambiguus*) and Captain (*Ara glaucogularis*), managed this in *critical test 2* after the first *short-tube experience* phase (Fig. 3; Supplementary Figs. 5 and 6 (OSM)). These two subjects only solved the task 11 times (rather than 12) before being moved onto the follow-up tasks due to experimenter error. Enya solved the task 11 times in 12 trials and Captain solved the task 11 times in 16 trials. All three of these subjects had four trials in which they obtained the reward without error, i.e. without inserting a counter-productive stone into the opposing end of the tube from their initial stone construction. Of these three, only Captain could consistently obtain the reward in the follow-up parallel tubes task, but he was unable to do so in the blocked tube task (Figs. 4 and 5).

None of the subjects were able to solve the task in the *pre-test* phase (Table 1; Fig. 3; Supplementary Figs. 1 and 2 (OSM)). Additionally, very few subjects inserted stones into the ends of the tube in this stage of the experiment (Supplementary Figs. 1 and 2 (OSM)). After the *stick experience* phase, in the first *critical test* phase, three of the *Ara glaucogularis* started to consistently insert stones into the ends of the tube (Fig. 3; Supplementary Fig. 3 (OSM)), whereas none of the *Ara ambiguus* did so at this stage (Fig. 3; Supplementary Fig. 4 (OSM)).

One more *Ara glaucogularis*, Mr Huang, had a few successful trials in *critical test 2*, but was unable to repeat this behaviour consistently (Supplementary Fig. 5 (OSM)). Of the eight *Ara ambiguus* that failed, six were unable to complete the *short-tube experience*, both before and after the *pre-inserted stone experience,* so they were never able to continue on to *critical test 2* (Table 1). The other two *Ara ambiguus* failed at *critical test 2* (Supplementary Fig. 6 (OSM)). Of the six *Ara glaucogularis* that failed, two were unable to complete the *short-tube experience* (both before and after the *pre-inserted stone experience;* Table 1) and the remaining four stopped at *critical test 2* (Supplementary Fig. 5 (OSM)). Thus in total, five *Ara glaucogularis* and three *Ara ambiguus* reached *critical test 2*. All the birds that attempted *critical test 2* inserted multiple stones into the apparatus, but most failed because they tried to insert them from both sides of the tube (Fig. 3; Supplementary Figs. 5 and 6 (OSM)).

There was a decrease in the number of times the subjects placed stones in the vertical tube on top of the apparatus over the *pre-test*, *critical test* and *critical test 2* (Supplementary Fig. 8 (OSM)). All but two birds inserted stones into this top tube in at least one trial in the *pre-test*, and many inserted stones in more than one trial.

Example videos of every phase of the experiment, including failed and successful trials of the critical tests, can be found on figshare (O’Neill & von Bayern, 2020).

## Discussion

Three subjects (two *Ara glaucogularis* and one *Ara ambiguus*) were able to consistently manufacture a multi-stone construction to obtain the walnut reward. These birds were able to create a functional stone-construction by combining multiple stone components one behind the other to push the walnut out of the tube. Due to their successes coming at different stages of the experiment, and because of some of the behaviours associated with their successful manufacture, it is likely that these successful subjects had a different understanding of their stone constructions and innovated them in different ways.

Lady (*Ara glaucogularis*) manufactured her first successful stone construction in *critical test 1* after having the functional experience that a stick tool could push the reward out of the tube. At this stage of the experiment, she had not had a specific positive experience that the stones could be used to obtain the reward in this specific apparatus (although she did have previous experience that putting singular stones into a different apparatus could lead to rewards; O’Neill et al., 2020). This kind of innovation suggests its possible she may have had some form of functional understanding of the stick tool example, and had positioned the stones to recreate its underlying purpose. On the other hand, it should be noted that Lady was also the only individual who consistently, and spontaneously, inserted stones into the horizontal tube in the *pre-test* (Fig. 3, Supplementary Figs 1 and 2 (OSM)), prior to any functional experience examples. Thus it is also possible that Lady did not need to learn anything functional from the *pre-inserted stick* experience in order to be successful. The *pre-inserted stick* experience may have just showed her that the walnut reward was obtainable, and thus motivated her to persist with the same exploratory behaviour of inserting stones that she had already shown in the *pre-test*.

Captain (*Ara glaucogularis*) and Enya *(Ara ambiguus*) were both able to consistently manufacture the stone construction in *critical test 2*, after they had the *short tube* functional experience. This experience showed the birds that a single stone was able to push a reward out of a much shorter horizontal tube. It was a much more direct example of the effect the stones could have on the rewards in the tube. Innovation of the stone construction at this stage of the experiment is much less likely to represent an innovation based on functional understanding. Instead, the birds are likely to have just been repeating the learned successful behaviour of inserting a single stone to get the reward, and this in turn led to them making the multi-stone construction, which eventually pushed the walnut out of the horizontal tube. The fact that all the subjects that took part in *critical test 2* began to insert multiple stones at this stage (Fig. 3) suggests that this was the underlying motivation to all the subjects’ behaviour at this stage. The difference with Captain and Enya is that for some reason they began to more consistently insert stones from a single side of the tube. This may have started initially as a side preference, but its possible that after multiple successes they began to more purposefully manufacture the tool from a single side only.

The three successful subjects each manufactured the multi-stone construction without errors on four occasions during their successful *critical test* runs. Out of context, the videos of these trials are compelling and can look fairly convincing that the subjects were building these stone constructions with ‘purpose’ to push the walnut rewards (see figshare at O’Neill & von Bayern, 2020, for videos). However, this interpretation should be considered with extreme caution. Captain was able to manufacture the stone construction without error on his first trial in *critical test 2* (Supplementary Fig. 5 (OSM); also shown in the supplementary videos) but subsequently produced many error-laden trials. Enya had an extremely error laden (but successful) first trial in *critical test 2*, but then immediately produced two ‘perfect’ trials in the minutes after this (trials 2 and 3, Supplementary Fig. 6 (OSM); also shown in the supplementary videos). She also subsequently produced many error-laden trials. Lady also shows similar behaviour of a mix of ‘perfect’ and error-laden trials (Supplementary Fig. 3 (OSM) and supplementary videos). The fact that these subjects only produced these ‘perfect’ trials erratically makes it very difficult to interpret why they sometimes manufacture the multi-stone construction without error, and sometimes they do not.

To an extent, all the successful subjects appeared to show an egocentric understanding (Woodward, 2011) of their behaviour, i.e. they were able to replicate it consistently and rapidly in trials following their initial success. They may have recognised which of their behaviours (repeatedly inserting stones from a single side of the horizontal tube) was producing the reward outcome. However, Captain did have one successful trial in *critical test 1* but was not able to replicate it at this stage of the experiment and Mr Huang (*Ara glaucogularis*) even had four successful trials early in *critical test 2*, but was then unable to replicate these successes. Neither of these failures appeared to be due to a lack of motivation as both were attempting to obtain rewards by inserting multiple stones into the horizontal tube, but they were just inserting them from both sides of the tube at once which led to the reward being trapped. It is unclear exactly which cue made the subjects recognise (or not recognise in Mr Huang’s instance) that the stones must be more consistently inserted from only a single side of the horizontal tube.

Regarding the successful birds, they made a number of mistakes when manufacturing their stone constructions. Each of the successful subjects only had four trials in their successful critical test in which they only inserted stones from a single side of the tube (Fig. 3; Supplementary Figs. 3, 5 and 6 (OSM)). On all their other trials (the majority), they inserted stones in both ends of the tube, thus on both sides of the reward. This type of ‘mistake’ is not just inefficient, but is counter-productive. It meant that more than the minimum number of stones had to be inserted in the original end of the tube to push out both the erroneous stones and then the reward. This suggests that the successful subjects did not have a mental representation of the final multi-stone tool that they were creating. This contrasts with a tool using corvid example, the New Caledonian Crow (*corvus moneduloides*), which were able to tear a piece of paper off a larger piece so that it resembled a token of the same size to that which they had previously learned could be traded for a reward, i.e. they could manufacture something that was similar to a template they had mentally stored (Jelbert et al., 2018). Observationally, the counter-productive stone insertion behaviour often happened after the subjects had manufactured an almost complete multi-stone construction from one side, then walked to the other side to check if they had pushed the reward far enough. When they could not reach the reward, they often inserted a stone into this other side of the tube that they were now standing next to (the supplementary videos provide examples of this error; see figshare at O’Neill & von Bayern, 2020, for videos). This may have been a self-control issue, as the birds would have had to walk back to the initial side of the tube in which they had already inserted stones in order to continue their stone construction already on the go. Instead, they were now standing closer to the reward, and it was likely difficult to inhibit repeating the stone insertion behaviour they had learnt led to a reward even though it pushed the reward further away from them again. Interestingly, this exact same mistake was observed in the original experiment that was run with capuchins (Visalberghi & Trinca, 1989). It is also an error that has been noted in other parrots taking part in an experiment with a very similar setup, the trap tube (Liedtke et al., 2011), and is something we had to control for when we tested a version of the trap tube on the same group of parrots tested in the current experiment (O’Neill et al., 2018).

This counter-productive stone insertion mistake was also observed in all of the unsuccessful subjects that were inserting stones into the tube. All of these unsuccessful subjects went through the *short-tube experience* where it is likely that they learned a simple heuristic rule of ‘insert stone, obtain reward’. Simply expanding this rule in a quantitative manner failed in *critical test 2* as the subjects had to ensure they inserted sufficient stones from a single side. It is thus possible that the successful birds did not have a different ‘rule’ to the other birds; they may have just had a weak side preference whereas the other subjects had no side preference at all. The three successful subjects (Enya, Lady and Captain) only collected the rewards from one side of the apparatus in all of the successful trials in the *critical tests*, i.e. they always built their stone construction from a specific side of the apparatus, they did not flexibly build it from either side.

Considering this side preference, it suggests why Lady and Enya failed in the *parallel tubes task,* in which only one tube was baited*.* Figure 4 shows that Lady was clearly inserting stones into both tubes (both the baited and unbaited tube), just from a specific side (also see supplementary videos at O'Neill & von Bayern, 2020). Although Enya was also mostly inserting stones from one side of the two tubes, her data are less convincing in supporting this side preference speculation.

Interestingly, Captain appears to have stopped having a side bias in this first *parallel tubes* follow-up task, and had successful trials in which he made his stone construction from both directions of the tube (Fig. 5; examples of success from both sides in supplementary videos at O'Neill & von Bayern, 2020). He also vastly improved the efficiency with which he made the multi-stone construction, exhibiting many trials in which he did not make the ‘mistake’ of inserting stones from both sides of the tube and he only inserted stones from one side of the tube to obtain the reward (11 out of 18 successful trials, although in four of those he also inserted stones into the un-baited tube as well). Observationally, one way that Captain behaved differently to Enya and Lady is that he showed a preference to stand on top of the apparatus when he was interacting with it, as opposed to the surface next to it. From personal observations, Captain was a more nervous bird. One behaviour the parrots manifested when they were nervous was try to stand on a higher perch and in this case the apparatus was the highest point in the testing room, hence he would stand on top of it. From this standpoint he had to move shorter distances to interact with the two ends of each tube, and was also able to transfer between the tubes very rapidly. Enya and Lady, on the other hand, had to walk ‘the long way round’ if they wanted to access different ends to the tubes. It’s possible that standing on top of the apparatus slightly reduced the self-control issues, as described above, which is what made Captain more efficient in creating the stone construction in the *parallel tubes* follow-up task.

Lady and Enya failed the *parallel tubes* transfer task, although it is possible that a lack of attention combined with lack of self-control (as discussed earlier), rather than a lack of means-end understanding of the stone construction, was the reason for this. Lady especially placed many stones in the un-baited ‘distractor’ tube and Enya especially began to place many stones in from both ends of the rewarded tube, although both made both errors at this stage. They both regularly ran out of stones. This behaviour suggests that Lady and Enya were probably not manufacturing their stone construction with the target to specifically push the reward, but that instead they likely had a more simple understanding of the apparatus, e.g. that inserting multiple stones would – at some point – make a reward appear. Further, it may be a reflection of the macaw’s relative lack of self-control to suppress a previously reinforced movement (Kabadayi et al., 2017), since they had never experienced a cost to inefficient stone inserting and had only been faced with tubes containing rewards previously. Limited use of the stone resources only became an issue in the transfer tasks, and therefore subjects may not have recognised that they could run out of them. Perhaps if they had been given more trials, they would have learnt to be more efficient and to pay attention where to insert the stones. Captain, on the other hand, was able to direct his multi-stone construction making behaviour towards the baited tube, which suggests he recognised that he had to target the stones towards the reward, not just the tubes in general. However, he was unsuccessful on the *blocked-tube* task, in which both tubes were baited but one had a blocked end. This suggests that while recognising the need to direct stones towards the reward, he did not grasp that the reward must be pushed to an accessible area, but only that the reward must be pushed. There were clearly limitations to the majority of the macaws overall understanding of the physical properties of this task.

In this test, an additional vertical tube was attached to the apparatus that had no effect on the reward outcome. This was to see if the subjects continued to use a basic heuristic of ‘*place stone on top of apparatus*’ to get the reward, as this was something that they had learnt led to success in a previous experiment (O’Neill et al., 2020; all except two subjects had learnt this behaviour (see Supplementary Table 2 in this paper)). Indeed, one of the first actions that almost all of the subjects did, in their initial exploration of the apparatus in the *pre-test*, was to insert a stone into this vertical tube (Supplementary Fig. 8 (OSM)). Only two subjects did *not* insert stones into the vertical tube in the *pre-test* and only one further subject did not place stones in this vertical tube in more than one trial. It could be argued that placing a stone in the pipe is evidence of a poor means-ends understanding of their actions and that their behaviour appeared to be guided more by trial-and-error exploration. However, not dropping stones into that tube would have required suppressing a previously reinforced behaviour and, thus, it should not be regarded as a clear-cut conclusion. Moreover, any exploration, even if it was misdirected and could not lead to success, helped the subjects learn more about the apparatus and its functional properties (Reader, 2015). The one subject that did not place stones in the top was the least explorative bird overall (he was the only bird to not make any combinatory actions between the stones and the horizontal tube (Supplementary Table 1)) and had no success in any of the current tasks (Hannibal), nor did he have any success in the previous task where the subjects had learned to drop a stone into a vertical tube (O’Neill et al., 2020). Further to this, as subjects did not place many stones into the sides of the horizontal tube in the pre-test but placed many stones in the vertical tube during the pre-test, it suggests that the subjects who eventually had success in the horizontal tube task did not succeed just because of carried-over behaviour from the success in the previous experiment. Thus, the most vital aspect to be learned from the addition of this vertical-tube was that subjects will try a previously used and successful method on a novel task before attempting something new.

Such a behavioural strategy, namely to exploit a similar feeding opportunity to one that is previously known, is thought to be a key mechanism characterising good innovators (Tebbich et al., 2016). The innovation structure described in Tebbich et al. (2016) provides a useful framework for how each subject’s behavioural interaction with the apparatus was possibly guided. As an example, the most successful subject, Captain, likely started the experiment with the knowledge from previous experimental apparatuses to ‘*insert stone in top*’ to access food. When this failed, he explored the apparatus and moved onto ‘*insert stones in tube ends’* (see Captain’s behaviour in the first *critical test*, Fig. 3), which he then newly discovered as a favourable interaction. He optimised this rule over successive interactions to discover that he had to ‘*insert stones from a specific side’* (see Captain’s behaviour in his successful *critical test 2*), and finally further optimisation led him to ‘*insert stones towards the reward from a single side’* (see Captain’s behaviour in the *parallel tubes* task)*.* All subjects appeared to go through this sort of heuristic ‘evolution’, but many of them never moved on from the first two stages. This suggests that almost all of the subjects were capable of recognising the feeding opportunity from the apparatus, and even discovering that there was a favourable behavioural interaction through exploration. What the majority of subjects struggled with was the repetition and testing required to optimise this favourable interaction to make exploiting this food as a worthwhile opportunity. A critical stage of the innovation framework (Tebbich et al., 2016) that is missing from our experiment is the opportunity to socially learn from other’s behaviour. If the other subjects could have watched Captain, Lady and Enya, perhaps they may have improved their interaction with the equipment. This is likely what would happen in the wild when conspecifics have discovered a novel feeding opportunity. Nevertheless, it is interesting that none of the subjects ever showed a truly flexible behaviour that would suggest a functional understanding of the underlying mechanics of the task, preferring always to instead rely on a simple rule or heuristic (Hutchinson & Gigerenzer, 2005). This suggests that these parrots are unlikely to use a complex mechanical causal understanding of these kinds of tasks (Johnson & Ahn, 2017; Woodward, 2011).

Overall, the subjects in this task showed less, or possibly zero, functional understanding of their multi-stone constructions when compared to other parrots’ functional understanding of tools they can use in problem-solving tasks (Auersperg et al. 2011; Auersperg, von Bayern, Gajdon et al. 2011, 2018; Auersperg et al., 2012; Laumer et al., 2017). As stated, the only individual that appeared to be using the stones in order to directly manipulate the position of the walnut was Captain, as he was the only one that would place the stones in the direction towards the walnut in the *parallel tubes* task. So although both species of macaw in this study appeared to show some evidence of innovative object manipulation in order to solve a problem-solving task, we still need to further study the differences between different parrot species (in this instance: Kea, Goffin’s cockatoos, *Ara glaucogularis* and *Ara ambiguus*) that make only some of them capable understanding the functional properties of more complex physical innovations.

## Supplementary Information

ESM 1 (DOCX 2716 kb)
