# Supplementary material for: Innovative problem solving in macaws
Source: Learn Behav. 2020 Dec 7;49(1):106–23. doi: 10.3758/s13420-020-00449-y (PMC7979646; doi:10.3758/s13420-020-00449-y)
Supplement: Supplementary file 1 — (DOCX 2716 kb) [file 13420_2020_449_MOESM1_ESM.docx]

Supplementary figure 1. Number of stones inserted in each side of the tube in the *pre-test* by the *Ara glaucogularis.*


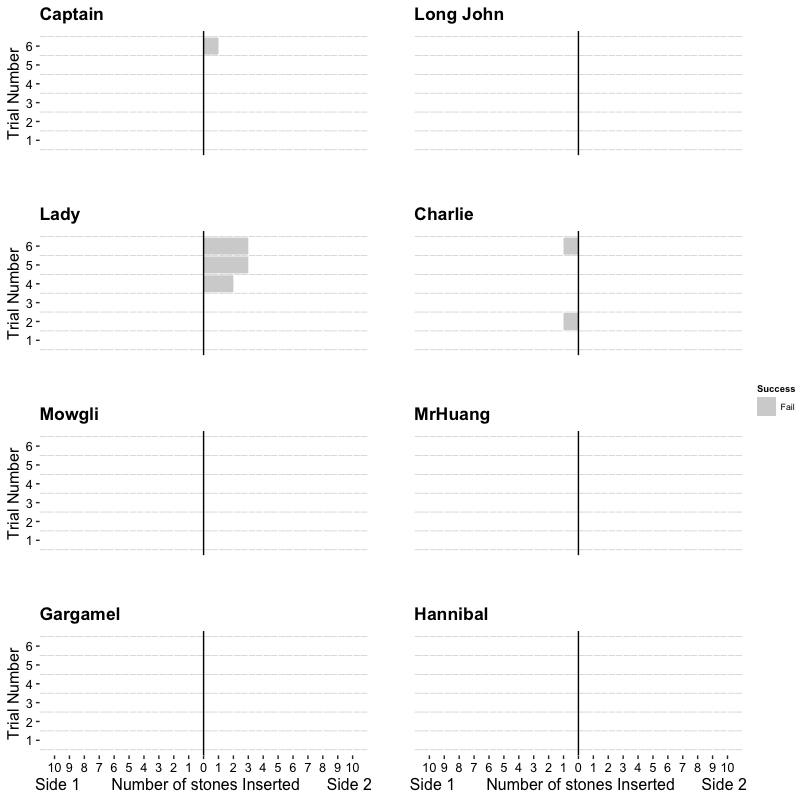
Supplementary figure 2. Number of stones inserted in each side of the tube in the *pre-test* by the *Ara ambiguus.*

*
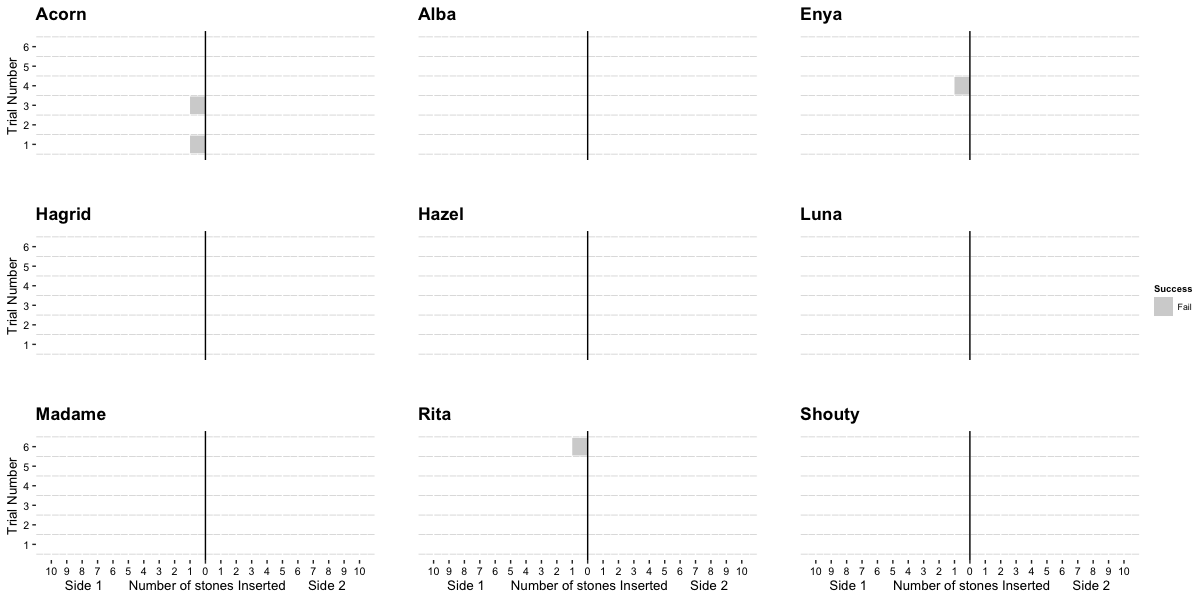
*Supplementary figure 3. Number of stones inserted in each side of the tube in the *critical test 1* by the *Ara glaucogularis.* Failed trials are shown in grey, successful ones in black. Lady reached the criterion of 12 successful trials in this phase, the majority of her stone insertions were biased to one side. For example, Charlie also inserted many stones, but from both sides of the tube so did not obtain the reward. Captain had a single successful trial but was unable to repeat this in following trials.


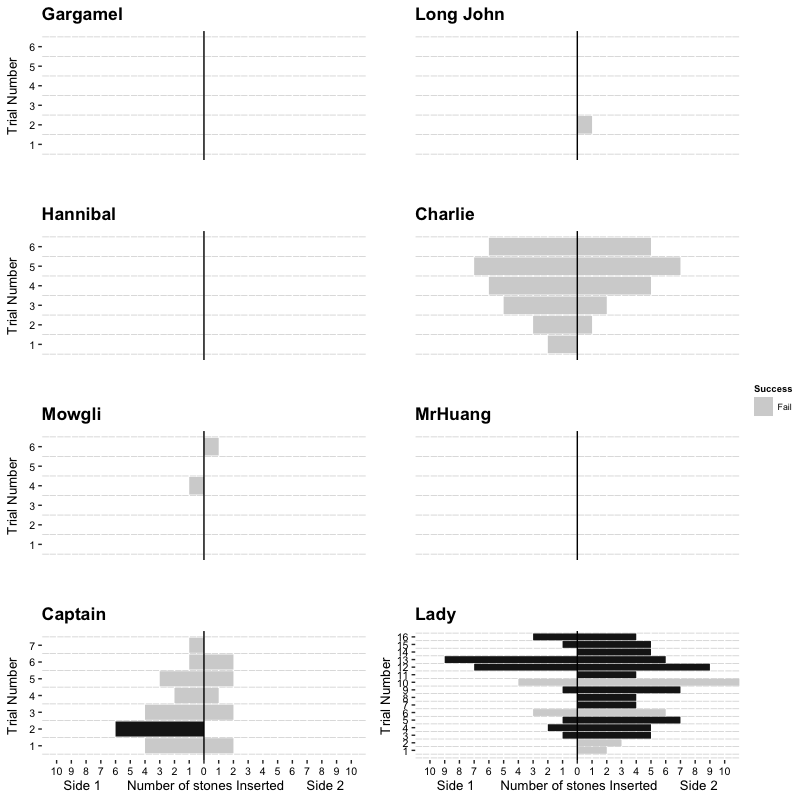
Supplementary figure 4. Number of stones inserted in each side of the tube in the *critical test 1* by the *Ara ambiguus.*


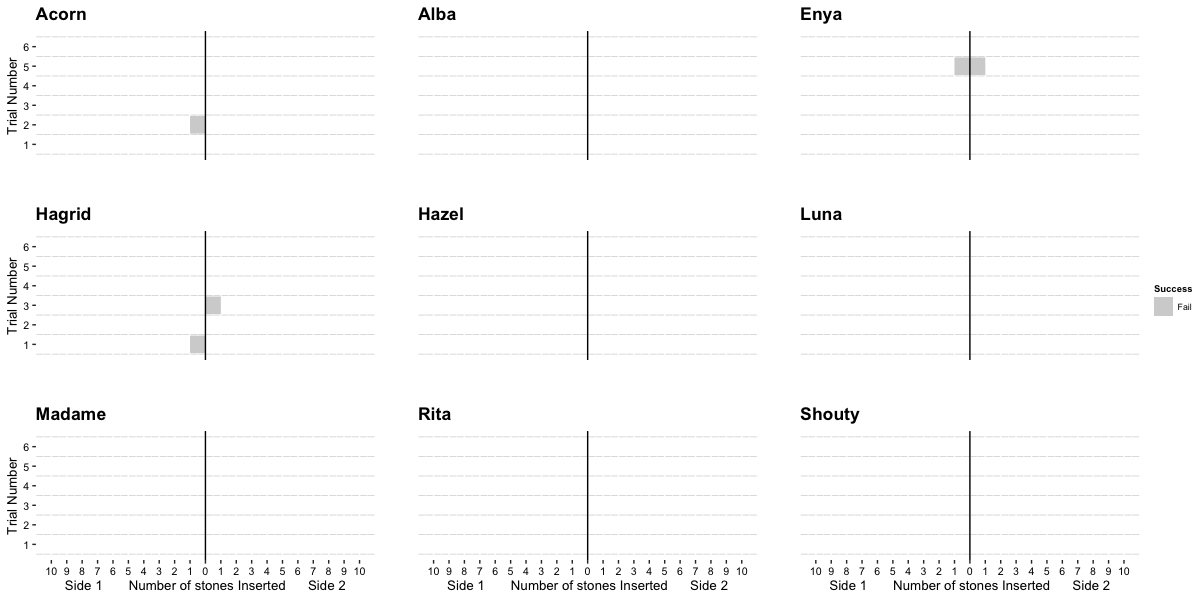
Supplementary figure 5. Number of stones inserted in each side of the tube in *critical test 2* by the *Ara glaucogularis.* Failed trials are shown in grey, successful ones in black. All of the subjects inserted many stones at this stage, but only Captain inserted the majority from a single side, thus obtaining the reward consistently.


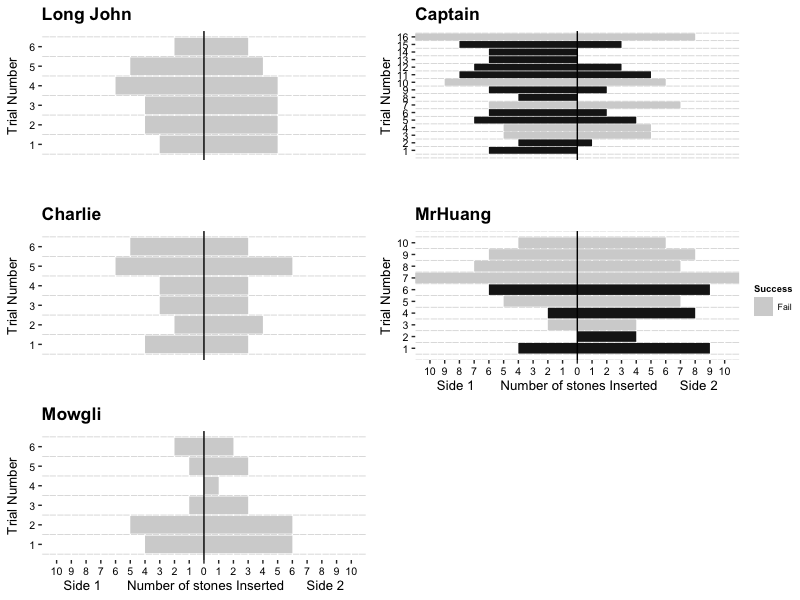

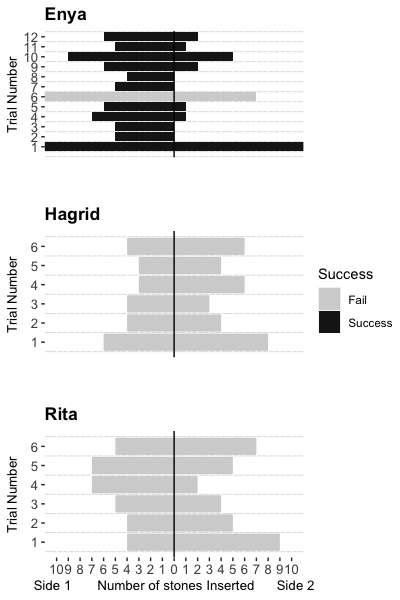


Supplementary figure 6. Number of stones inserted in each side of the tube in *critical test 2* by the *Ara ambiguus.* Not many of the *Ara ambiguus* succeeded in the *short tube experien*ce phase, hence only a few of the subjects reached second *critical test 2*. Only Enya succeeded in this phase. She notably inserted the majority of stones from one side of the tube. The other two individuals also started inserting many stones, but did so on each side.


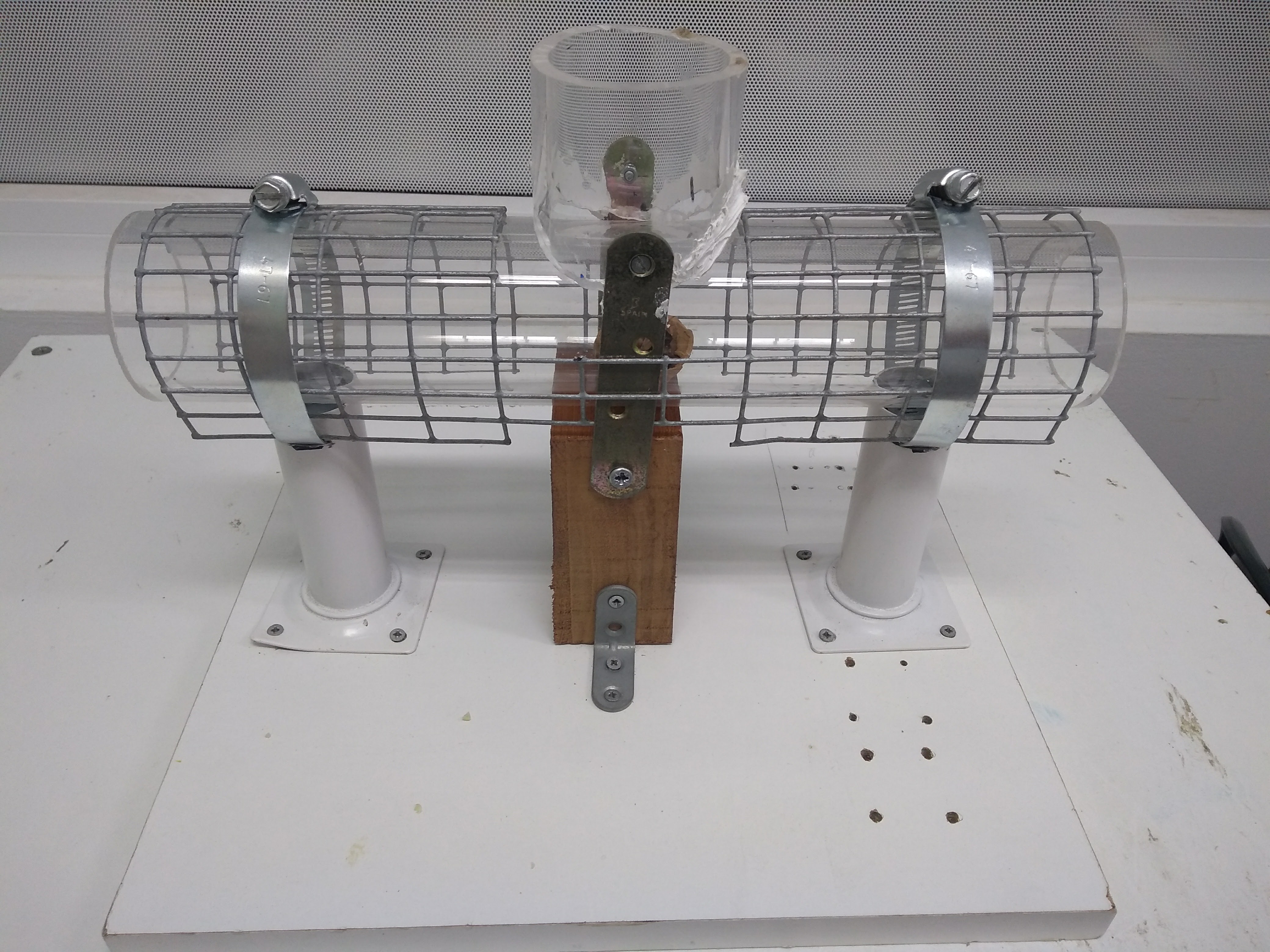


Supplementary figure 7. The apparatus used for the test phases of the experiment. Specifically, it shows the wire mesh surrounding the tube to show the ‘solidity’ of the transparent acrylic. Also, it shows the vertical tube attached to the top of the tube. This tube had no connection to the functional horizontal tube in any way, but just trapped inserted stones.


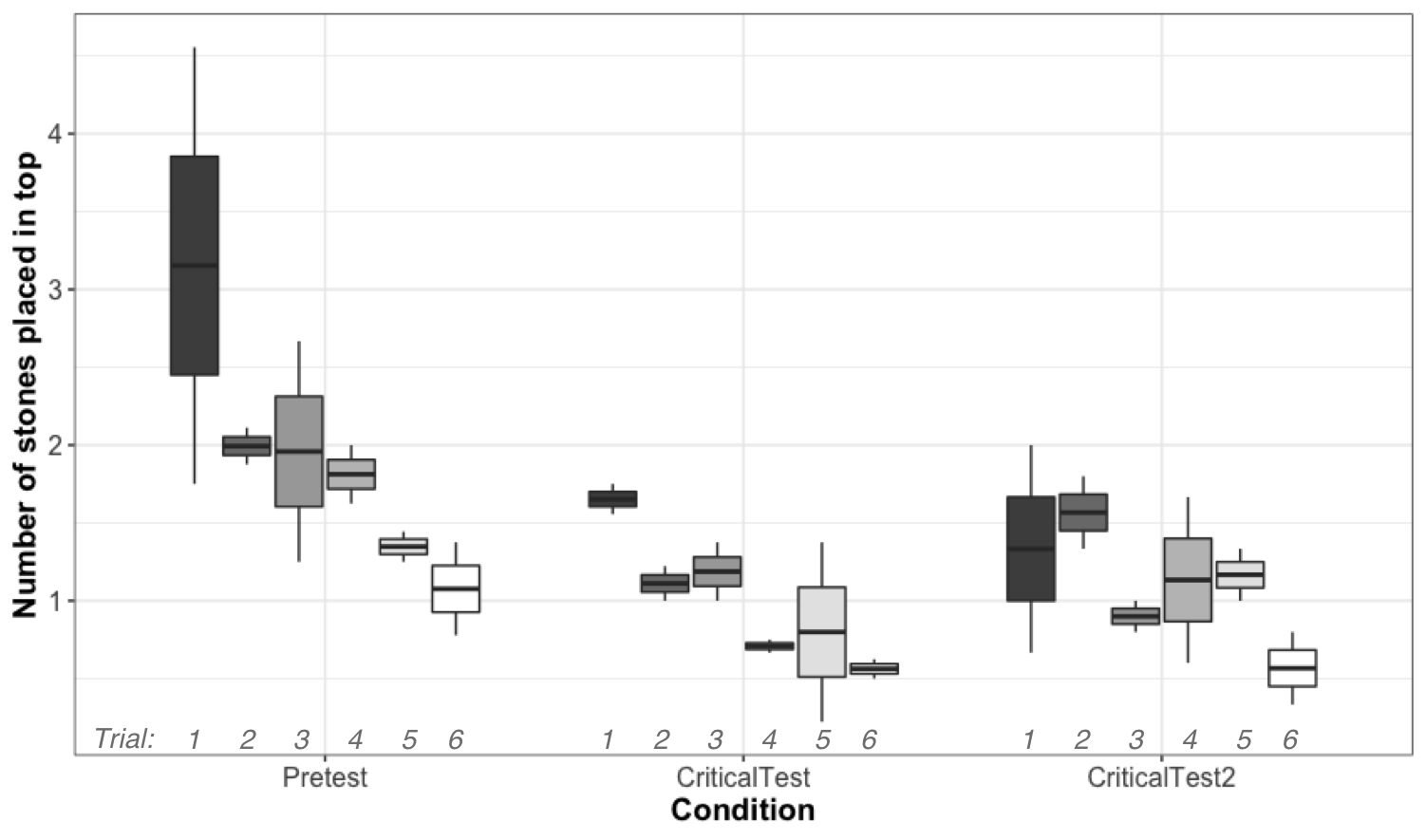


Supplementary figure 8. The mean number of times subjects inserted stones into the top tube in the different test conditions, divided into the first six trials of each condition. Their was a notable increase in the number of stones inserted into this vertical tube on the first trial of the pretest compared to all the following trials.


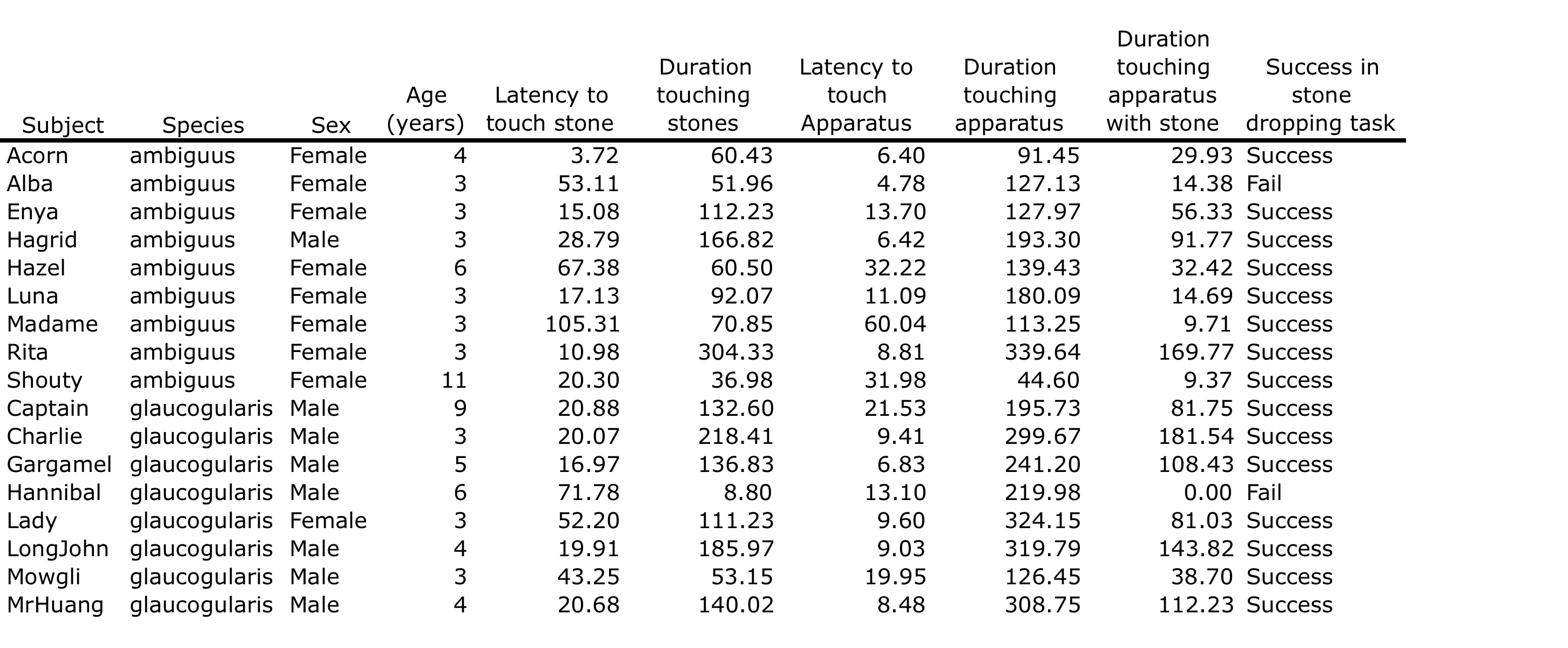
Supplementary Table 1. Description of subjects, their exploration amount of the horizontal tube apparatus and their success in an experiment prior to the horizontal tube task. The average (mean) exploration amounts (in seconds) are reported from the first trial in the pre-test up until subjects had their first successful trial, hence the exploration presented here is the subjects exploration amounts up until they were aware the test was solvable. The previous experiment all subjects took part in, the stone dropping task, required subjects to drop stones into a vertical tube onto a collapsible platform to release a reward. Almost all the subjects were able to solve this other task which gave them prior experience with stones as ‘tool-like’ objects.
